# Antiferroelectric PbSnO_3_ Epitaxial Thin Films

**DOI:** 10.1002/advs.202203863

**Published:** 2022-10-26

**Authors:** Yu‐Hong Lai, Jun‐Ding Zheng, Si‐Cheng Lu, Yin‐Kuo Wang, Chun‐Gang Duan, Pu Yu, Yun‐Zhe Zheng, Rong Huang, Li Chang, Ming‐Wen Chu, Ju‐Hung Hsu, Ying‐Hao Chu

**Affiliations:** ^1^ Department of Materials Science and Engineering National Yang Ming Chiao Tung University Hsinchu 30010 Taiwan; ^2^ Key Laboratory of Polar Materials and Devices Ministry of Education Department of Electronics East China Normal University Shanghai 200241 China; ^3^ State Key Laboratory of Low Dimensional Quantum Physics and Department of Physics Tsinghua University Beijing 100084 China; ^4^ Center for General Education National Taiwan Normal University Taipei 10610 Taiwan; ^5^ Center for Condensed Matter Sciences and Center of Atomic Initiative for New Materials National Taiwan University Taipei 106 Taiwan; ^6^ Integrated Service Technology Hsinchu 300 Taiwan; ^7^ Center for Emergent Functional Matter Science National Yang Ming Chiao Tung University Hsinchu 30010 Taiwan; ^8^ Department of Materials Science and Engineering National Tsing Hua University Hsinchu 30013 Taiwan

**Keywords:** antiferroelectricity, epitaxial thin films, functional oxides, lead stannate, perovskites

## Abstract

In condensed matter physics, oxide materials show various intriguing physical properties. Therefore, many efforts are made in this field to develop functional oxides. Due to the excellent potential for tin‐based perovskite oxides, an expansion of new related functional compounds is crucial. This work uses a heteroepitaxial approach supported by theoretical calculation to stabilize PbSnO_3_ thin films with different orientations. The analyses of X‐ray diffraction and transmission electron microscopy unveil the structural information. A typical antiferroelectric feature with double hysteresis and butterfly loops is observed through electrical characterizations consistent with the theoretical prediction. The phase transition is monitored, and the transition temperatures are determined based on temperature‐dependent structural and electrical characterizations. Furthermore, the microscopic antiferroelectric order is noticed under atomic resolution images via scanning transmission electron microscopy. This work offers a breakthrough in synthesizing epitaxial PbSnO_3_ thin films and comprehensively understanding its anisotropic antiferroelectric behavior.

## Graphical abstract

The epitaxial PbSnO_3_ (PSO) thin films with different orientations are stabilized by pulsed laser deposition. Through electrical measurement, the typical antiferroelectricity of PSO can be confirmed. In addition, the antiferroelectric order of PSO is unveiled by scanning transmission electron microscopy. This work offers an appealing approach in synthesizing PSO and deeply explores its characteristics.

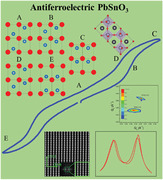

## 1 Introduction

Complex perovskite oxides show various unique properties, including superconductivity,^[^ ^1^ ^]^ magnetism,^[^ ^2^, ^3^ ^]^ colossal magnetoresistance,^[^ ^4^ ^]^ piezoelectricity,^[^ ^5^, ^6^ ^]^ multiferroicity,^[^ ^7^, ^8^ ^]^ etc. In most cases, the functionality is driven by the strong correlation in transition elements. Less attention was paid to other groups of elements due to the lack of d electrons. Recently, the progress of tin‐based perovskite oxides has drawn much attention. For example, La‐doped BaSnO_3_ can reach high mobility of 300 cm^2^V^−1^s^−1^ as transparent conducting oxide.^[^ ^9^, ^10^ ^]^ Except for the high mobility, the 2D electron gas detected at the interface between BaSnO_3_/LaInO_3_ delivers a vast potential for next‐generation oxide electronics.^[^ ^11^ ^]^ Besides, the discovery of ferroelectric (FE) ZnSnO_3_ (47 µC cm^−2^) based on FeTiO_3_ ilmenite structure delivers a potential candidate for non‐volatile random access memory.^[^ ^12^, ^13^ ^]^ These fascinating properties make tin‐based oxides a great potential for application in touch panels, solar cells, and transparent electronics. Therefore, the development and improvement of tin‐based perovskite oxide represent an important research field. To trigger more functionalities in tin‐based perovskite oxides, a typical way to adjust the physical properties of perovskite oxides is the replacement of ions. Replacing A‐site cations with different radii or valence electron configurations can change the ground state and show completely different properties. Due to the presence of 6s lone pair electrons in various perovskite oxides, Pb ion commonly exists in A‐site, such as PbZrO_3_ (PZO) and PbTiO_3_ (PTO), resulting in antiferroelectricity and ferroelectricity.^[^ ^14^, ^15^, ^16^ ^]^ Therefore, it is straightforward to consider the feasibility of exploring PbSnO_3_ (PSO), and it is anticipated to discover distinct properties which have never been observed in other tin‐based perovskite oxides.

In the evaluation of the structural stability, the value of Goldschmidt tolerance factor $t=\left(r_{\mathrm{Pb}}+r_{O}\right)/\sqrt{2}\left(r_{\mathrm{Sn}}+r_{O}\right)$, is used to estimate the distortion and stability of perovskite of PSO,^[^ ^17^, ^18^ ^]^ where *r*_Pb_, *r*_Sn_ and *r*_O_ are the ionic radii of Pb, Sn and O ions, respectively. In this case, the calculated value of *t* is 0.88 for PSO, implying the stability of PSO. In addition, the standard enthalpy of PSO formation is negative theoretically, suggesting the stable existence of PSO single phase.^[^ ^19^ ^]^ Despite the above reasons pointing to the stable presence of PSO, single crystal PSO with perovskite structure is still challenging to be synthesized. In the experimental part, the PSO compound's dominant structure is pyrochlore rather than perovskite.^[^ ^20^ ^]^ To synthesize perovskite PSO, it must be carried out under extremely high pressure.^[^ ^21^ ^]^ Besides the difficulties in synthesizing perovskite PSO, it is also difficult to control the PSO configuration. According to past studies, nanoparticles or clusters with polycrystalline PSO have been chemically synthesized in most experiments.^[^ ^20^, ^22^ ^]^ However, the irregular structure causes specific problematic issues in characterizing physical properties. Besides, it cannot acquire information on anisotropy from polycrystalline samples. Due to these issues, PSO's electrical performance and anisotropy remain unexplored. Hence, to understand the characteristics of PSO, it is necessary to fabricate high‐quality single‐crystal PSO thin films.

In this study, a heteroepitaxy approach is adopted with the control of growth, and epitaxial PSO thin films can be fabricated on SrTiO_3_ (STO) substrates with SrRuO_3_ (SRO) bottom electrode via pulsed laser deposition.^[^ ^23^ ^]^ Taking advantage of the form of epitaxial thin films, it is feasible to measure their physical properties and the corresponding anisotropic behaviors. In the electrical measurement, epitaxial PSO films show a distinct antiferroelectric (AFE) feature with a transition temperature of 150 °C. Besides, the AFE order is unveiled by atomic resolution images. This work explores the corresponding physical characteristics to reveal the antiferroelectricity of PSO and expands the functionality of tin‐based perovskite oxides.

## 2 Results and Discussions

**Figure 1:**
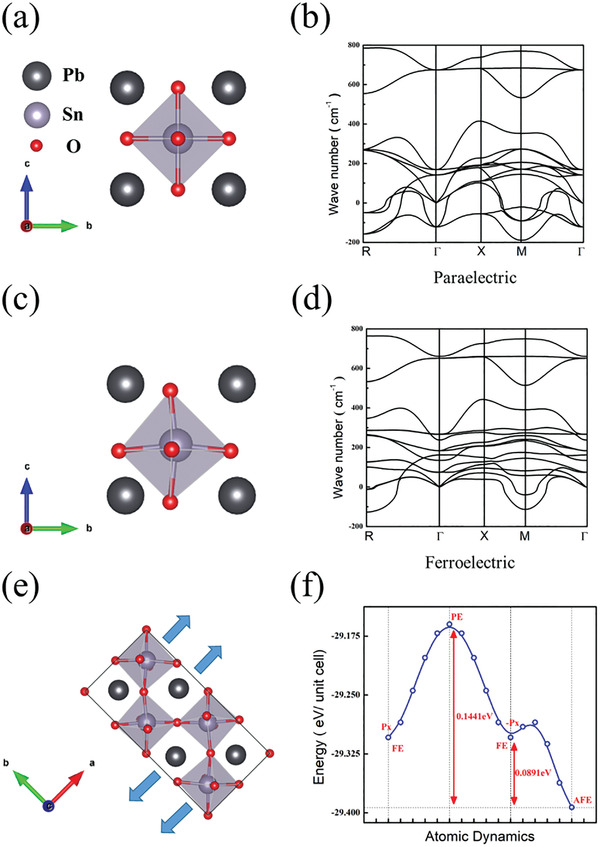
Density function theory (DFT) calculations and unit cells of PSO structure. a) Unit cell of PE PSO (cubic, *a* = 4.115 Å, unit‐cell volume = 69.68 Å^3^). b) Phonon spectrum of PE PSO. c) Unit cell of FE PSO (cubic, *a* = 4.115 Å, unit‐cell volume = 69.68 Å^3^). d) Phonon spectrum of FE PSO. e) Unit cell of AFE PSO (orthorhombic, *a* = 5.7954 Å, *b* = 11.5909 Å, *c* = 8.3682 Å, unit‐cell volume = 562.12 Å^3^). f) The potential profile of PSO from DFT calculations. The blue arrows represent the polarization direction.

The density function theory (DFT) calculations were carried out to understand the PSO characteristics to obtain the phonon spectrum. As shown in **Figure** **1**a,b, the imaginary frequencies of the paraelectric (PE) phase at symmetry point R(1/2, 1/2, 1/2), Γ(0, 0, 0) and M(1/2, 1/2, 0), suggest that the paraelectric PSO with cubic phase is unstable. In ABO_3_ perovskite materials, the Γ point is inconsistent FE mode, the R point represents the antiphase tilting mode of oxygen atoms about [100] and [010] axis, and the M point represents the in phase tilting mode about the [001] axis.^[^ ^24^ ^]^ The oxygen octahedral tilting is usually termed antiferrodistortive mode (AFD).^[^ ^25^, ^26^ ^]^ In addition, the Σ point (1/4, 1/4, 0) is a critical mode, which is related to the displacement of A‐site cation along the [110] direction.^[^ ^14^, ^27^ ^]^ Combined with its wave vector, it represents AFE distortion. According to the simple two‐instability Landau‐type theory, there must be of at least two soft lattice modes with a close instability temperature for the emergence of the AFE phase.^[^ ^28^, ^29^ ^]^ and the condition is satisfied in PSO. To further analyze the properties of the PSO, we soften the Γ mode by giving a shift to the Sn atom, breaking the lattice symmetry, and then relaxing the structure. Finally, we obtain the FE structure. It should be emphasized here that although it is usually the Pb atom distorted due to the presence of lone‐pair electrons, moving Pb or Sn atoms are equivalent for a single cell. The structure and phonon spectrum of the FE phase are shown in Figure 1c,d. Due to the introduction of ferroelectricity, the Γ mode is completely softened. Then, the part of the AFD modes at R and M points disappear due to the tilting of oxygen atoms, but there are still some remaining modes, and there is no sign of softening. Finally, as the relative displacement of the Pb atom, the Σ mode disappears. Interestingly, the Σ mode softens earlier than the Γ mode, suggesting that the AFE transition temperature (*T*_A_) is close to but higher than the Curie temperature (*T*_c_), which is the condition for AFE two‐instability Landau‐type theory. Therefore, we extend the unit cell to the orthonormal supercell to increase the degree of freedom. On this basis, the AFE structure shown in Figure 1e is obtained after relaxation. The AFD is expressed as a^−^a^−^c^+^ by Glazer's sign.^[^ ^25^ ^]^ In the AFD mode, the oxygen atom is antiphase tilting along the [100] and [010] axes and in phase titling along the [001] axis. The A‐site cation undergoes AFE distortion at the [110] axis.^[^ ^24^ ^]^ Additionally, when Goldschmidt tolerance factor *t* < 0.9, the crystal structure tends to have the tilting of oxygen octahedron.^[^ ^30^, ^31^ ^]^ In PSO, *t* = 0.88, which further increases the credibility of the existence of the AFD mode AFE structure. In Figure 1f, we calculated the potential profile of FE‐PE‐FE‐AFE, among which the AFE has the lowest energy, so we can speculate that there is an AFE phase in PSO.

It is also worth noting that the frequency of acoustic modes is suppressed with the formation of the ferroelectric phase, that is, the softening of the specific optical mode in the paraelectric phase. The phenomenon is derived from the flexoelectric coupling between the optic and the acoustic modes. The researchers pointed out that flexoelectric coupling could lead to the formation of an incommensurate phase.^[^ ^32^ ^]^ When analyzing the origin of PZO AFE, it is reported that the appearance of the commensurate AFE phase in pure PZO prevents the system from entering the incommensurate phase.^[^ ^14^ ^]^ Still, there are incommensurate states in modified PZO or other similar materials.^[^ ^33^, ^34^, ^35^ ^]^ We infer that the phenomenon also exists in PSO; PSO crystal could produce an incommensurate state with the effect of domain walls or substrates.

**Figure 2:**
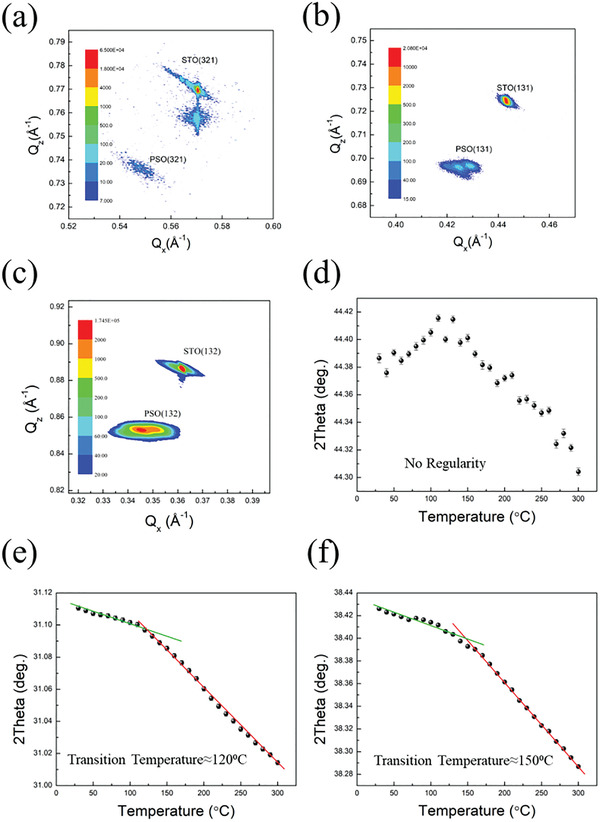
Structural information of the PSO films. RSMs of a) the [100]_PSO_ sample with *Q*_z_ = [100]_STO_ and *Q*_x_ = [021]_STO_, b) the [110]_PSO_ sample with *Q*_z_ = [110]_STO_ and *Q*_x_ = [$\bar{1}$11]_STO_, and c) the [111]_PSO_ sample with *Q*_z_ = [111]_STO_ and *Q*_x_ = [$\bar{1}$10]_STO_. Theta‐2 theta scan of d) [100]_PSO_ sample, e) [110]_PSO_ sample, and f) [111]_PSO_ sample from room temperature to 300 °C.

To verify the theoretical prediction, PSO thin films were deposited via pulsed laser deposition on STO substrates under the same growth condition. An investigation of the orientation effect is employed based on the samples with three different orientations, whose out‐of‐plane directions are [100]_PSO_, [110]_PSO_ and and [111]_PSO_ separately. In the following discussion, a pseudocubic system is adopted to illustrate the structure and index of PSO. The phase identification was performed by X‐ray diffraction (XRD). From the results of the theta‐2 theta scan, the single series of out‐of‐plane PSO peaks were observed without detecting any other secondary phase, as shown in Figures S1a to S1c, Supporting Information. In addition, the scan of Phi rotation was applied to these three samples to examine the in‐plane symmetry, as shown in Figure S1d, Supporting Information. For the [100]_PSO_ sample, the (111) plane was detected four times with a 90° interval while rotating 360° around the out‐of‐plane direction, which is consistent with the fourfold symmetry of the pseudocubic {100} plane. Based on the single out‐of‐plane direction and the symmetric in‐plane scanning, the heteroepitaxy of the [100]_PSO_ sample is confirmed. For the same reasons, the heteroepitaxy of [110]_PSO_ and [111]_PSO_ samples can be inferred, as shown in Figures S1e and S1f, Supporting Information. Transmission electron microscopy (TEM) was carried out on these three samples with various orientations to examine the microstructure of the PSO samples. In the high‐resolution images taken from the [111]_PSO_ sample along the [$\bar{1}\bar{1}2$] zone axis, the PSO/SRO and SRO/STO interfaces are prominent and sharp, indicating high‐quality heterostructure, as shown in Figure S2, Supporting Information. Furthermore, the corresponding fast Fourier transform (FFT) shows clear spots at each layer. The index of these spots can be determined based on the assignment of the reciprocal lattice, as shown in the inset of Figure S2, Supporting Information. These clear spots further verify the epitaxy and exhibit the superior crystallinity of PSO thin film. Besides, since these layers of the films show similar FFT patterns, it can infer that the structures of PSO will be identical to SRO and STO. From the TEM results of [110]_PSO_ and [100]_PSO_ samples, the same conclusions can be drawn according to the results from the [111]_PSO_ sample, as shown in Figures S3 and S4, Supporting Information. The consistency is confirmed compared to the XRD and TEM results, and the epitaxial relationship can be defined, as shown in Figure S5, Supporting Information. After the fundamental understanding of the structure, the next object is to acquire the lattice parameters of PSO films, the reciprocal space mappings (RSMs) were carried out at room temperature, as shown in **Figure** **2**a–c. From these results, the lattice parameters of the [100]_PSO_ sample (*a* = 0.4080 nm, *b* = 0.4068 nm, *c* = 0.4047 nm, *α* = *γ* = 90°, and *β* = 89.5°), the [110]_PSO_ sample (*a* = 0.4048 nm, *b* = 0.4069 nm, *c* = 0.4095 nm, *α* = *γ* = 90°, and *β* = 89.5°), and the [111]_PSO_ sample (*a* = 0.4106 nm, *b* = 0.4085 nm, *c* = 0.3962 nm, *α* = *γ* = 90°, and *β* = 89.5°) can be obtained. This result matches the previous research accordingly.^[^ ^17^ ^]^ After a thorough establishment of the structural information at room temperature, attention was paid to the search for phase transition of the PSO films. The two theta‐temperature relationships of each sample were recorded from room temperature to 300 °C, as shown in Figure 2d–f. Based on these results, it is noticed that the lattice constants tend to increase with temperature due to thermal expansion. More importantly, the change of slope can also be detected, which usually corresponds to a phase transition, at ≈120 °C in the [110]_PSO_ sample and 150 °C in the [111]_PSO_ sample. However, a similar phenomenon was not observed in the [100]_PSO_ sample. Therefore, we can reasonably infer that the [100]_PSO_ sample may show different physical properties compared to the samples of [110]_PSO_ and [111]_PSO_ in the following measurement. Since the change of slope was discovered, it has been able to calculate the thermal expansion coefficient (*α*) before and after phase transition. The formula used here is *α* = (Δ*L*)/*L*_0_ × (Δ*T*), where Δ*L* is the change of length, *L* is the initial length and Δ*T* is the change of temperature. The calculated values *α* are 4.04 × 10^−6^ and 1.46 × 10^−5^ for the [110]_PSO_ sample and 5.66 × 10^−6^ and 1.88 × 10^−5^ for the [111]_PSO_ sample in the regime of low and high temperatures, respectively. It is worth noting that the value of *α* in the high‐temperature regime is three times larger than the one in the low‐temperature regime, which is independent of orientations, suggesting the consistent existence of low and high‐temperature phases in the [110]_PSO_ and [111]_PSO_ samples.

**Figure 3:**
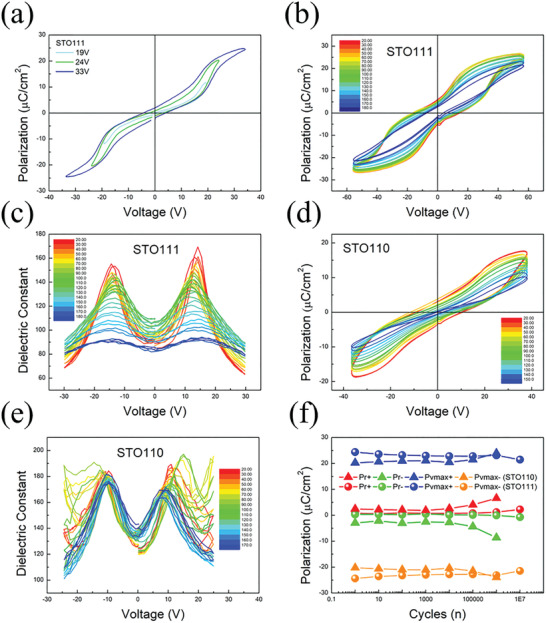
*P*–*V*, *C*–*V*, and reliability measurements. a) The *P*–*V* loop of the [111]_PSO_ sample with increasing voltage to saturation. b) *P*–*V* measurement of [111]_PSO_ sample from room temperature to 180 °C with frequency *f* = 400 kHz. c) *C*–*V* measurement of [111]_PSO_ sample from room temperature to 180 °C with frequency *f* = 1 MHz. d) *P*–*V* measurement of [110]_PSO_ sample from room temperature to 150 °C with frequency *f* = 300 kHz. e) *C*–*V* measurement of [110]_PSO_ sample from room temperature to 170 °C with frequency *f* = 1 MHz. f) The Pr+, Pr−, Pvmax+, and Pvmax− values of two oriented PSO films were extracted from *P*–*V* loops as a function of the cycling number at room temperature.

After exploring the theoretical and structural parts, the next object focuses on the electrical characterization of these samples. The differently oriented samples performed the polarization–voltage (*P*–*V*) and capacitance–voltage (*C*–*V*) measurements. As shown in **Figure** **3**a and Figure S6, Supporting Information, the double hysteresis loops were obtained on the [111]_PSO_ and [110]_PSO_ samples to show the saturated polarization (*P*_max_) of ≈25 and ≈20 µC cm^−2^, respectively. However, for the [100]_PSO_ sample, the results show a straight line, typical paraelectricity. This observation represents that only the [111]_PSO_ and [110]_PSO_ samples exhibit a clear AFE property. These results are consistent with the XRD results. In this part, we speculate that the different behavior of the three oriented samples comes from the subtle differences in structure. In the RSM measurement, the structure is defined as monoclinic, which is close to cubic practically. The [100]_PSO_ sample's structure is very close to cubic with no asymmetric center inside, resulting in a paraelectric behavior. In contrast, the structure of the [110]_PSO_ and [111]_PSO_ samples are monoclinic with an asymmetric center inside, which can explain their antiferroelectricity. Based on this inference, the cubic phase is more likely to be a high‐temperature phase compared to the monoclinic phase, which can explain the existence of phase transition observed in [110]_PSO_ and [111]_PSO_ samples but [100]_PSO_ sample. As shown in Figure 2d, while considering the temperature ranging from 110 to 300 °C, the *α* of [100]_PSO_ is ≈1.08 × 10^−5^, which is close to the high‐temperature phase of [110]_PSO_ and [111]_PSO_ samples. Therefore, it can reasonably explain the missing antiferroelectricity in the [100]_PSO_ sample. Furthermore, the *P*–*V* and *C*–*V* measurements with temperature dependence were performed on the [110]_PSO_ and [111]_PSO_ samples from 20 to 180 °C, as shown in Figure 3b–e. The *P*–*E* characteristic curve gradually transforms from an AFE double loop to a paraelectric characteristic during the heating process. Based on these results, it can assume that the transition temperature is ≈120 °C for the [110]_PSO_ sample and 150 °C for the [111]_PSO_ one, which is similar to the results of temperature‐dependent XRD measurement. In addition, the dielectric constant–temperature curves at various frequencies, which are commonly reliable in obtaining the phase transition temperature, are measured. As shown in Figure S7, Supporting Information, we found the temperature‐dependent dielectric constant of the PSO films. The transition temperatures of the [110]_PSO_ and [111]_PSO_ samples are close to ≈120 and ≈150 °C measured at 1 MHz. These values match the transition temperature of the PSO films from the AFE phase to the single‐cubic phase. Therefore, it can conclude the consistency in determining the transition temperature. According to research, the transition temperature can be tuned by fabricating films on different substrates and using the clamping effect. For instance, the *T*_c_ of PZO can be lowered from 232 (bulk) to 220 °C (thin film) via depositing on MgO substrates.^[^ ^36^ ^]^ In addition, the *T*_c_ of PZO thin film can be slightly increased by fabricating on STO substrates, which induce compressive strain.^[^ ^37^ ^]^ We deposited a thick film (>300 nm) to minimize the substrate clamping effect, and the substrate orientation was used to modify surface energy to stabilize the PSO perovskite phase.

For the *C*–*V* measurement, the double‐butterfly curve, which shows a further split compared to ferroelectric curves, is exhibited at 20 °C (red lines in Figure 3c–e). During the heating, the split of the left and right peaks gradually approaches and forms a single peak, which means the disappearance of antiferroelectricity. Therefore, the tendency of the *C*–*V* curves and *P*–*V* curves is consistent. Moreover, it is noticed that the curves are not the same. As we know, the *C*–*V* curves come from the differentiation of the *P*–*V* loops. The difference between these two *C*–*V* curves originates from the two *P*–*V* loops. In Figure 3b–d, the [111]_PSO_ sample shows a clear ferroelectric‐to‐antiferroelectric transition. Therefore, a more obvious split of left and right peaks can be observed on the [111]_PSO_ sample. In contrast, the [110]_PSO_ sample does not show a clear ferroelectric‐to‐antiferroelectric transition. The split of left and right peaks is not apparent in the [110]_PSO_ sample. At the left and right endpoints of the two *C*–*V* curves, the [111]_PSO_ sample presents more consistent values than the [110]_PSO_ sample, which is also a difference. We can find this difference at low temperatures in the temperature‐dependent *C*–*V* measurement. After passing the transition temperature, the difference is eliminated. Besides, it is noticed that the measurement frequency plays an important role at different temperatures in the *P*–*V* measurement. Therefore, the *P*–*V* characterization was performed on the [111]_PSO_ sample with frequency dependence during the heating process. As shown in Figure S8, Supporting Information, the *P*–*E* loop recorded with 50 kHz exhibits the most excellent performance compared to other frequencies at 20 °C. However, the 50 kHz loop breaks down when the temperature reaches 110 °C. In contrast, the 400 kHz loop can sustain stably up to 110 °C despite its worse performance at 20 °C. Besides, the leakage current of the 400 kHz loop is even lower at higher temperatures, which can be inferred from the polarization difference at 0 V. Therefore, the most suitable operating frequency can vary depending on temperature. The assumption about this phenomenon may come from the thermal instability at high temperatures. For the solid solution of PSO‐based perovskite, it is reported that its structure undergoes a phase transition from AFE phase to multi‐cell cubic (MCC) phase to single cubic phase. This similar situation has a great opportunity to occur in PSO thin films. At high‐temperature regimes, such as 110 °C, the AFE and MCC phases coexist in PSO thin films.^[^ ^38^, ^39^ ^]^ In the *P*–*V* measurement, the randomly arranged domain wall between these two phases causes some irreversible motion and makes the hysteresis loop break down. Hence, it is the reason to adopt higher frequency at high temperatures to freeze the motion of the domain wall. After understanding the dependence of orientation, temperature and frequency, the next object explores the reliability issues of PSO thin films. The fatigue measurements were performed on the samples of [110]_PSO_ and [111]_PSO_, as shown in Figure 3f. PSO thin films can tolerate up to 10^6^ cycles for the [110]_PSO_ sample and 10^7^ cycles for the [111]_PSO_ sample at room temperature without obvious failure, indicating the certain stability of PSO. In the development of AFE materials, a dielectric capacitor for energy storage is an attractive research direction due to their great potential for power electronics. Thus, attention was paid to investigating the performance of energy storage. The energy storage efficiency (*η*) and the energy storage density (*W*_d_) are noteworthy points. For the 50 kHz loop of the [111]_PSO_ sample recorded at 20 °C, *η* and *W*_d_ can reach 75% and 19.8 J cm^−3^. Compared to epitaxial PZO, PbHfO_3_ (PHO) and AgNbO_3_ (ANO) films, as shown in **Table** **1**, our epitaxial PSO films can tolerate a high electric field (1950 kV cm^−1^). Due to this reason, PSO has a high value of *W*_d_, which is higher than PZO (*W*_d_ = 12.5 J cm^−3^) and ANO (W_d_ = 5.8 J cm^−3^). Besides, the *η* of PSO is higher than PHO (*η* = 69%) and ANO (*η* = 55.8%). Therefore, the PSO AFE system has a great opportunity to be applied to energy storage devices. However, certain explorations should be further made to optimize the performance.

**Table 1:** A comparison of PSO and other AFE epitaxial thin films

	PbSnO_3_ (this work)	PbZrO_3_^[^ ^40^ ^]^	PbHfO_3_^[^ ^41^ ^]^	AgNbO_3_^[^ ^42^ ^]^
Pvmax+ [µC cm^−2^]	20 (110) 25 (111)	32 (100) 43 (110) 45 (111)	31 (100) 39 (110) 33 (111)	43 (100)
Pr+ [µC cm^−2^]	3 (110) 2 (111)	1 (100) 3 (110) 3 (111)	4 (100) 2 (110) 2 (111)	9 (100)
Electric field [kV cm^−1^]	1950 (111)	700 (100) 700 (110) 700 (111)	1250 (100) 1300 (110) 1250 (111)	625 (100)
Energy density [J cm^−3^]	19.8 (111)	7.4 (100) 12.2 (110) 12.5 (111)	16.5 (100) 21.5 (110) 15.5 (111)	5.8 (100)
Efficiency [%]	75 (111)	X	54 (100) 69 (110) 60 (111)	55.8 (100)
Fatigue (cycles)	10^6^ (110) 10^7^ (111)	X	10^9^ (100) 10^9^ (110) 10^9^ (111)	X

**Figure 4:**
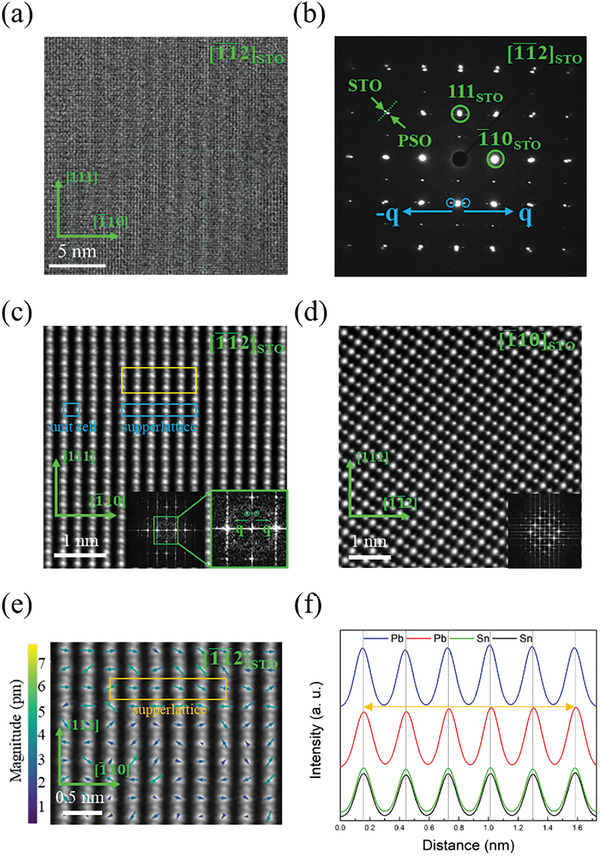
Analyses of AFE order. a) PSO high‐resolution TEM image was taken along [$\bar{1}\bar{1}$2]_STO_ with in‐plane direction [$\bar{1}$10]_PSO_. b) SAED pattern of PSO heterostructure. Incommensurate vectors [$\bar{1}$10]*5.5 (± q, marked with blue circles) are unveiled. c) STEM‐HAADF image of PSO taken along [$\bar{1}\bar{1}$2]_STO_, revealing the incommensurate modulation (± q, marked with green circles). The yellow rectangle represents the superlattice doubled along [111] (blue rectangles, unit cell, and primitive superlattice). d) STEM‐HAADF image of PSO taken along [$\bar{1}10$]_STO_. The incommensurate vectors (± q) in Figure 4c become invisible under this projection, parallel to ± q. e) Displacement of Pb ion (marked with arrows) with Sn as the reference frame (orange rectangle, superlattice), accounting for the superlattice modulation. f) Profiling the Pb and Sn positions in (e). Vertical gray lines, positions of the Sn above and beneath the Pb considered (red curve: the Pb position in orange rectangle, blue curve: the first Pb row below the red curve). The double arrow orange line marks the size of the superlattice.

Due to the reason that the AFE behavior mentioned above is a macroscopic phenomenon, it is necessary to provide the AFE order of the PSO thin film from a microscopic viewpoint to be more convincing. Considering the direct relation between AFE order and atomic displacement, high‐angle annular dark‐field scanning transmission electron microscopy (HAADF‐STEM) is a powerful method for analyzing PSO thin films. **Figure** **4**a shows the cross‐sectional high‐resolution TEM image of [111]_PSO_ sample taken along [$\bar{1}\bar{1}$2]_STO_. The noticeable stripes in the image usually correspond to the modulated period, which is the AFE modulation in this case. From the selected area electron diffraction (SAED) pattern, the incommensurate [$\bar{1}$10] × 5.5 is observed, as shown in Figure 4b. The split in the prominent diffraction spot comes from the different lattice parameters of STO and PSO, whose boundary is indicated by a green dotted line. In real space, the modulated superlattice corresponds to 15.65 Å. Figure 4c shows a typical HAADF image taken along [$\bar{1}\bar{1}$2]_STO_ zone axis. Because the contrast of the HAADF image is roughly proportional to the square of the atomic number (*Z*), the brighter and larger ion is Pb, and the other is Sn. The atomic arrangement of PSO agrees with the AFE PSO structure in the part of the theoretical calculation, as shown in Figure S9, Supporting Information. Moreover, it can be observed that the center of Pb and the Sn ions are not aligned along the same straight line, which is the origin of antiferroelectricity. As shown in Figure S10, Supporting Information, in the area enclosed by a yellow rectangle in Figure 4c, the center asymmetry of Pb and Sn ions can be more clearly noticed. The position of ions at each horizontal row will be discussed in the following. From the FFT shown in the inset of Figure 4c, it is worth noting that the incommensurate [$\bar{1}$10] × 5.5 appears in the horizontal direction, which is consistent with the findings in Figure 4b. Besides, the modulated period disappears while rotating the zone axis in Figure 4c to 90° along the in‐plane direction along [$\bar{1}$10], as shown in Figure 4d. According to the past study performed on AFE PHO thin films,^[^ ^41^ ^]^ the results report that the origin of its antiferroelectricity comes from the displacement of Pb atoms with the frame composed of Hf atoms. Therefore, we took the Sn ions as the frame and compared the displacement of Pb ions according to the Sn frame. In this case, we used atomap,^[^ ^43^ ^]^ a Python library, to analyze the Pb displacement. By fitting the 2‐D Gaussian function to every atomic column in Figure 4c, the Sn‐lattice and Pb‐lattice can be constructed, as shown in Figure S11, Supporting Information. The spontaneous Pb displacement can be determined by analyzing the shift of the Pb‐lattice to that of the Sn‐lattice. As shown in Figure 4e, most of the Pb‐displacement range from 3 to 6 pm. It is found that the vertical component of Pb displacement aligns randomly along the modulation vector. The random arrangement of vertical components comes from the dense arrangement of Pb and Sn ions along [111], which causes difficulties in analysis. Therefore, attention should be paid to the horizontal component. Considering the horizontal component of Pb displacement, they align in the same direction along the modulation vector and the opposite direction perpendicular to the modulation vector. Hence, it can be concluded that the PSO possesses an AFE order. In Figure 4f, the line profile of Sn (green and black curves) and Pb (red and blue curves) are extracted from the yellow rectangle in Figure 4c. Compared to the Sn‐lattice, the displacement of the Pb‐lattice can be estimated to be ≈6.5 pm, which is comparable to the analysis in Figure 4e. Refer to the common FE compounds, the saturation polarization (*P*) can be estimated according to *P* = (258 ± 9)Δ*Z* *µ*C cm^‐2^, where Δ*Z* is an average displacement.^[^ ^44^ ^]^ Therefore, the estimated *p* value is ≈17 µC cm^−2,^ which is close to the results of the *P*–*E* measurement on the [110]_PSO_ sample (20 µC cm^−2^).

## 3 Conclusion

In summary, the PSO thin films were fabricated epitaxially on STO substrates with three different orientations. The structural information has been well studied, and the epitaxial relationship was established based on the results of XRD and TEM. The temperature‐dependent XRD measurement determined the phase transition temperatures to be 120 °C for the [110]_PSO_ sample and 150 °C for the [111]_PSO_ sample. All these results mentioned above are consistent with past research. The *P*–*V* and *C*–*V* measurements confirmed the antiferroelectricity in the PSO thin films with superior reliability. In addition, the evidence of AFE order is observed via the HAADF‐STEM technique directly. In this work, the fundamental characteristics of PSO are thoroughly investigated. Based on this deep understanding, PSO can serve as a promising candidate for improving the AFE energy storage device and has an excellent opportunity to be the main component in other modern electronics.

## 4 Experimental Section

### Sample Preparation

The PSO target comprises the PbO and SnO_2_ powders with a ratio equal to 1.1:1. The excess amount of PbO ensures the improvement of Pb evaporation during processing. The STO substrates were cleaned beforehand in alcohol and acetone ultrasonically and attached to a SiC‐made heater with a silver paste. The growth condition was 115 mtorr and 640 °C for PSO, and the cooling rate was 10 °C min^−1^. For Pt top electrode, it was deposited at room temperature with a circular pattern (*r* = 50 µm) by the radio‐frequency sputter method.

### DFT Calculation

Fully‐relativistic density functional theory calculations were performed using Quantum Espresso (QE). The Perdew–Burke–Ernzerhof exchange–correlation functional has been used in structural relaxation. The kinetic energy cutoff was 40 Ry and the charge density cutoff of 160 Ry, and Monkhorst–Pack k‐point grids (5 × 5 × 5) were adopted. The energy tolerance was 10^−6^ Ry. The Hellmann–Feynman forces on each atom were less than 10^−4^ Ry/a.u. The phonon Brillouin zone was sampled with Q‐points translated from 3 × 3 × 3 mesh for the unit cell.

### XRD

The theta‐2 theta scan along the out‐of‐plane direction was performed with a Bruker D2 PHASER X‐ray Powder Diffractometer. The phi scan was served with a Bruker D8 DISCOVER SSS Multi Function High Power X‐Ray Diffractometer. The wavelength of the Cu X‐ray was 0.15406 nm. The RSM and temperature‐dependent XRD were characterized by a high‐resolution four‐circle X‐ray diffractometer (Smartlab, Ragaku).

### Electrical Measurement

The *P*–*V* and *C*–*V* measurement were performed by TFAnanlyzer3000, aix ACCR Systems. The AC frequency and alternating voltage were 100 mV and 10 kHz for *P*–*V* measurement and 1 V and 1 MHz for *C*–*V* measurement, respectively.

### TEM

The cross‐sectional TEM specimens were fabricated using the focused ion beam technique in the FEI Helios G4 system, including a low‐energy polishing process at 5 and 2 keV. The TEM and STEM analyses were carried out on a double spherical aberration (Cs) corrected JEM‐ARM300F microscope operated at 300 kV. A probe convergence semiangle of 18 mrad and a detection semiangle larger than 64 mrad were used for the HAADF observations. All STEM images were filtered in Fourier space using Gatan digital micrograph to remove the high‐frequency noise.

## Conflict of Interest

The authors declare no conflict of interest.

## Supporting information

Supporting Information

## Data Availability

The data that support the findings of this study are available in the supplementary material of this article.
