# Supplementary material for: Antiferroelectric PbSnO3 Epitaxial Thin Films
Source: Adv Sci (Weinh). 2022 Oct 26;9(35):2203863. doi: 10.1002/advs.202203863 (PMC9762279; doi:10.1002/advs.202203863)
Supplement: Supplementary file 1 — Supporting Information [file ADVS-9-2203863-s001.pdf]

ADVANCED SCIENCE
Open Access

Supporting Information

for *Adv. Sci.*, DOI 10.1002/advs.202203863

Antiferroelectric $PbSnO_3$ Epitaxial Thin Films

Yu-Hong Lai, Jun-Ding Zheng, Si-Cheng Lu, Yin-Kuo Wang, Chun-Gang Duan, Pu Yu, Yun-Zhe Zheng, Rong Huang, Li Chang, Ming-Wen Chu, Ju-Hung Hsu and Ying-Hao Chu**

Supporting Information

Antiferroelectric $PbSnO_3$ Epitaxial Thin Films

Yu-Hong Lai, Jun-Ding Zheng, Si-Cheng Lu, Yin-Kuo Wang, Chun-Gang Duan, Pu Yu, Yun-Zhe Zheng, Rong Huang, Li Chang, Ming-Wen Chu, Ju-Hung Hsu, Ying-Hao Chu**

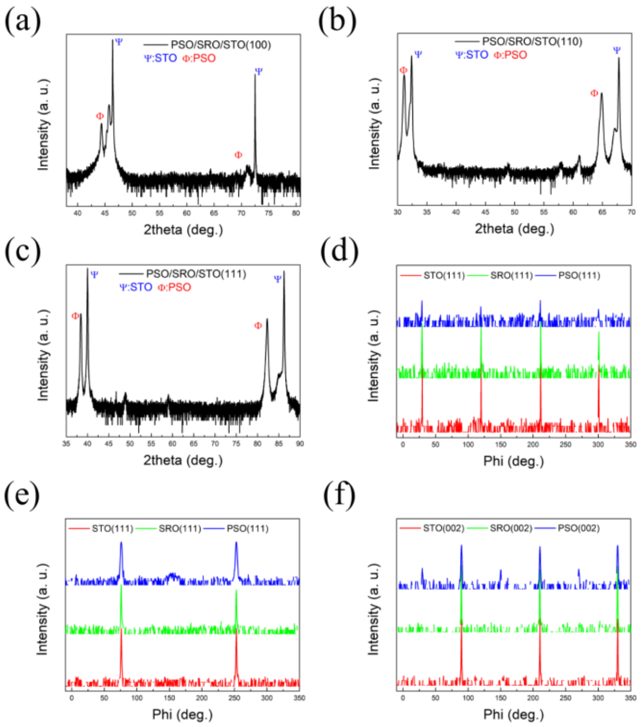

Figure S1. Theta-2 theta scan of the (a) $[100]_{PSO}$ sample, (b) $[110]_{PSO}$ sample and (c) $[111]_{PSO}$ sample. Phi scan of the (d) $[100]_{PSO}$ sample, (e) $[110]_{PSO}$ sample and (f) $[111]_{PSO}$ sample.

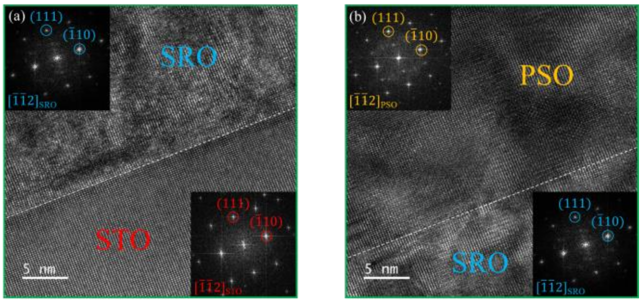

Figure S2. High-resolution images were taken at (a) SRO/STO (b) PSO/SRO interfaces of the $[111]_{PSO}$ sample.

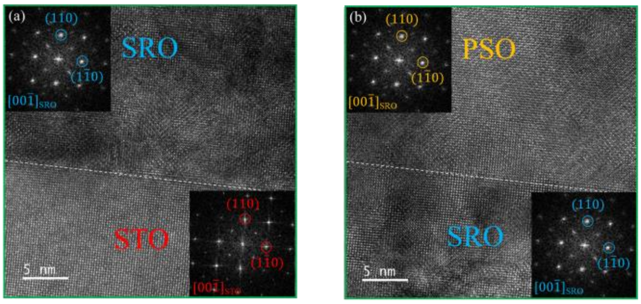

Figure S3. High-resolution images were taken at (a)SRO/STO and (b) PSO/SRO interfaces of the $[110]_{PSO}$ sample.

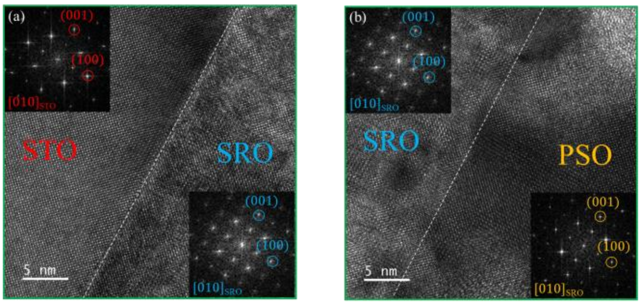

Figure S4. High-resolution images were taken at (a)SRO/STO and (b) PSO/SRO interfaces of the $[100]_{PSO}$ sample.

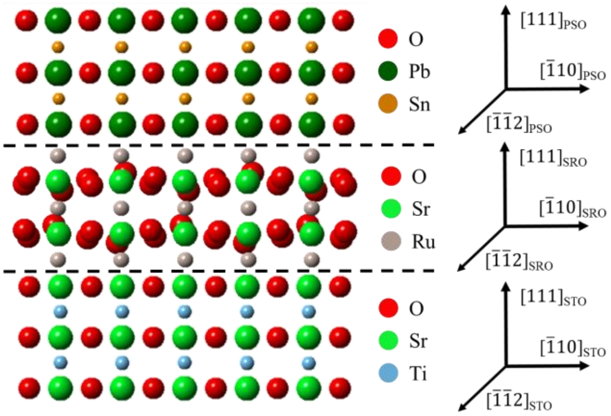

Figure S5. Epitaxial relationship of the PSO film with SRO/STO.

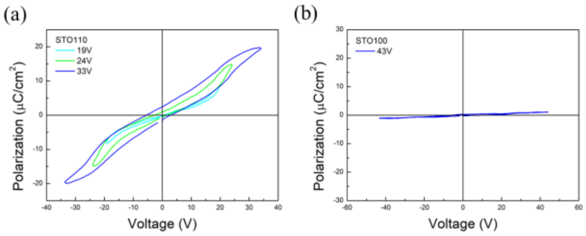

Figure S6. The P-V loops of (a) $[110]_{PSO}$ and (a) $[100]_{PSO}$ samples.

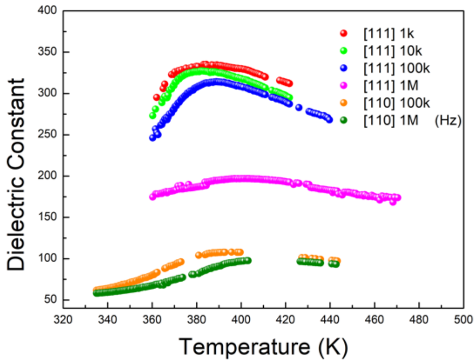

Figure S7 Temperature-dependent dielectric constants of the PSO films at various frequencies.

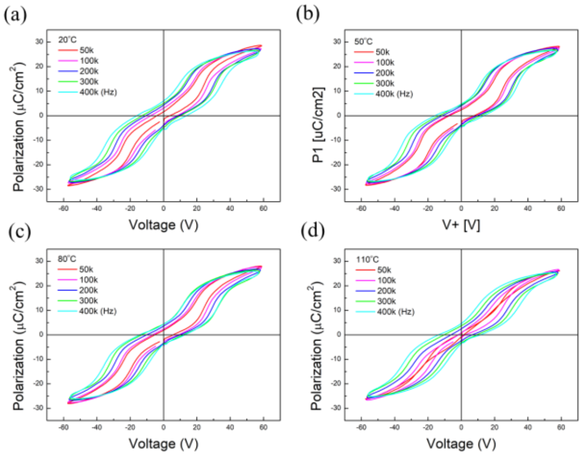

Figure S8. The P-E loops of $[111]_{PSO}$ sample with temperature dependence. (a) 20℃ (b) 50℃ (c) 80℃ (d) 110℃.

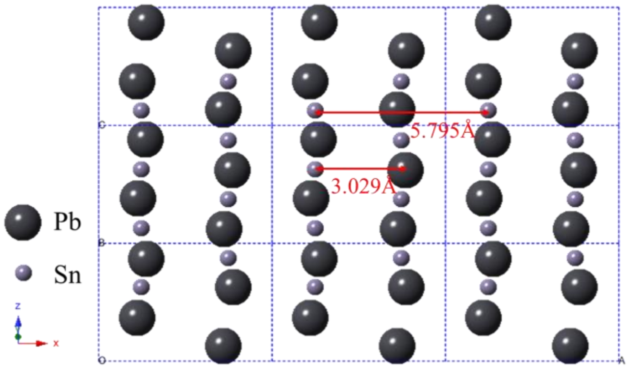

Figure S9. The atomic arrangement of AFE PSO with zone axis $= [\bar{1}\bar{1}2]_{STO}$.

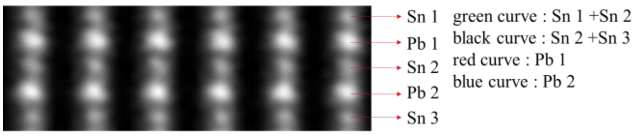

Figure S10. The yellow rectangle encloses the enlarged image in Figure 4c. The line profile of each horizontal row with different colors is shown in Figure 4f.

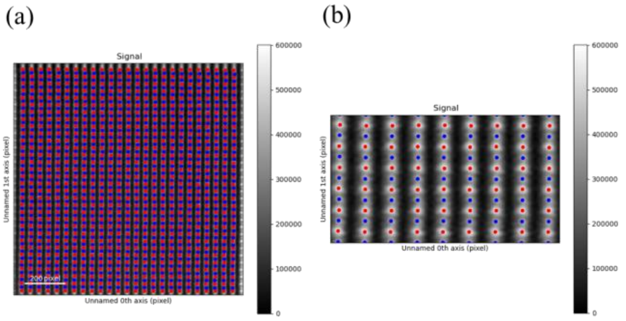

Figure S11. The Sn-lattice (blue dots) and Pb-lattice (red dots) of the PSO HAADF image. (a) whole image (b) zoom-in image of (a).
